# Resolution of pituitary microadenoma after coronavirus disease 2019: a case report

**DOI:** 10.1186/s13256-021-03127-3

**Published:** 2021-11-01

**Authors:** Salah Raishan, Mohammed Alsabri, Ann Mary Hanna, Matthew Brett

**Affiliations:** 1 Emergency Medicine Department, Al Thawra Modern General Hospital (TMGH), Sana’a City, Yemen; 2 College of Osteopathic Medicine, NYIT, Glen Head, New York, USA; 3 Brookdale University Hospital and Medical center, 1 Brookdale Plaza, Brooklyn, NY 11212 USA

**Keywords:** COVID-19, Pituitary microadenoma, Spontaneous resolution, Case report

## Abstract

**Background:**

This report describes the case of a patient whose pituitary microadenoma resolved after he contracted coronavirus disease 2019. To our knowledge, this is one of the first reported cases of pituitary tumor resolution due to viral illness. We present this case to further investigate the relationship between inflammatory response and tumor remission.

**Case presentation:**

A 32-year-old man in Yemen presented to the hospital with fever, low blood oxygen saturation, and shortness of breath. The patient was diagnosed with coronavirus disease 2019. Past medical history included pituitary microadenoma that was diagnosed using magnetic resonance imaging and secondary adrenal insufficiency, which was treated with steroids. Due to the severity of coronavirus disease 2019, he was treated with steroids and supportive care. Three months after his initial presentation to the hospital, brain magnetic resonance imaging was performed and compared with past scans. Magnetic resonance imaging revealed changes in the microadenoma, including the disappearance of the hypointense lesion and hyperintense enhancement observed on the previous scan.

**Conclusions:**

Pituitary adenomas rarely undergo spontaneous resolution. Therefore, we hypothesized that tumor resolution was secondary to an immune response to coronavirus disease 2019.

## Background

Pituitary adenomas are benign tumors of the anterior pituitary that are true neoplasms according to the results of clonality studies. They can arise from any type of cell of the anterior pituitary and may result in increased secretion of the hormone(s) produced by those cells and/or decreased secretion of other hormones owing to a reduction in other cell types. Pituitary adenomas are the most common cause of sellar masses from the third decade of life, accounting for up to 10% of all intracranial neoplasms [1]. Adenomas are classified according to both size and cell of origin; lesions < 1 cm are classified as microadenomas, whereas lesions > 1 cm are classified as macroadenomas. Sellar masses can present with neurologic symptoms, abnormalities related to under- or over-secretion of pituitary hormones, or may be incidental findings on radiologic examinations performed for other reasons. In a nonfunctioning adenoma, impaired vision is a common symptom for which patients seek medical attention. Patients often present with headaches due to the expansion of the sella and diplopia [2]. Many patients with pituitary adenomas present with signs and symptoms of hormone hypersecretion (hyperprolactinemia, growth hormone excess, or hypercortisolism). However, 25–35% of the tumors are clinically nonfunctioning or “silent” [3]. Patients with pituitary macroadenomas can develop serious complications, such as pituitary apoplexy (PA), massive internal hemorrhage, and pituitary necrosis [4]. PA is caused by infarction, hemorrhage, or hemorrhagic infarction, and leads to the compression of nearby neurological structures. The symptoms include sudden onset of headache, visual impairments, potentially altered mental status, and sensory or motor dysfunction. Hemorrhage occurs more frequently in macroadenomas because of high vascularity or in nonfunctioning adenomas. Spontaneous disappearance of the tumor following PA is rarely observed. Spontaneous remission of endocrinopathy and regression of the tumor following an apoplectic event is a well-known phenomenon in cases of hormonally active pituitary adenomas; however, the resolution of a nonfunctioning adenoma following PA is rare [5]. Only 13 cases, including the present case, of spontaneous resolution of nonfunctional pituitary adenomas have been reported in 26 years [6].

This case study may provide insights into the implementation of immuno-CoV-2 cancer vaccine therapeutic strategies to co-target tumors and COVID-19, as well as support cellular immunity to make hosts resistant to tumor development via inoculation with tumor site-guided agents.

## Case presentation:

**Fig. 1:**
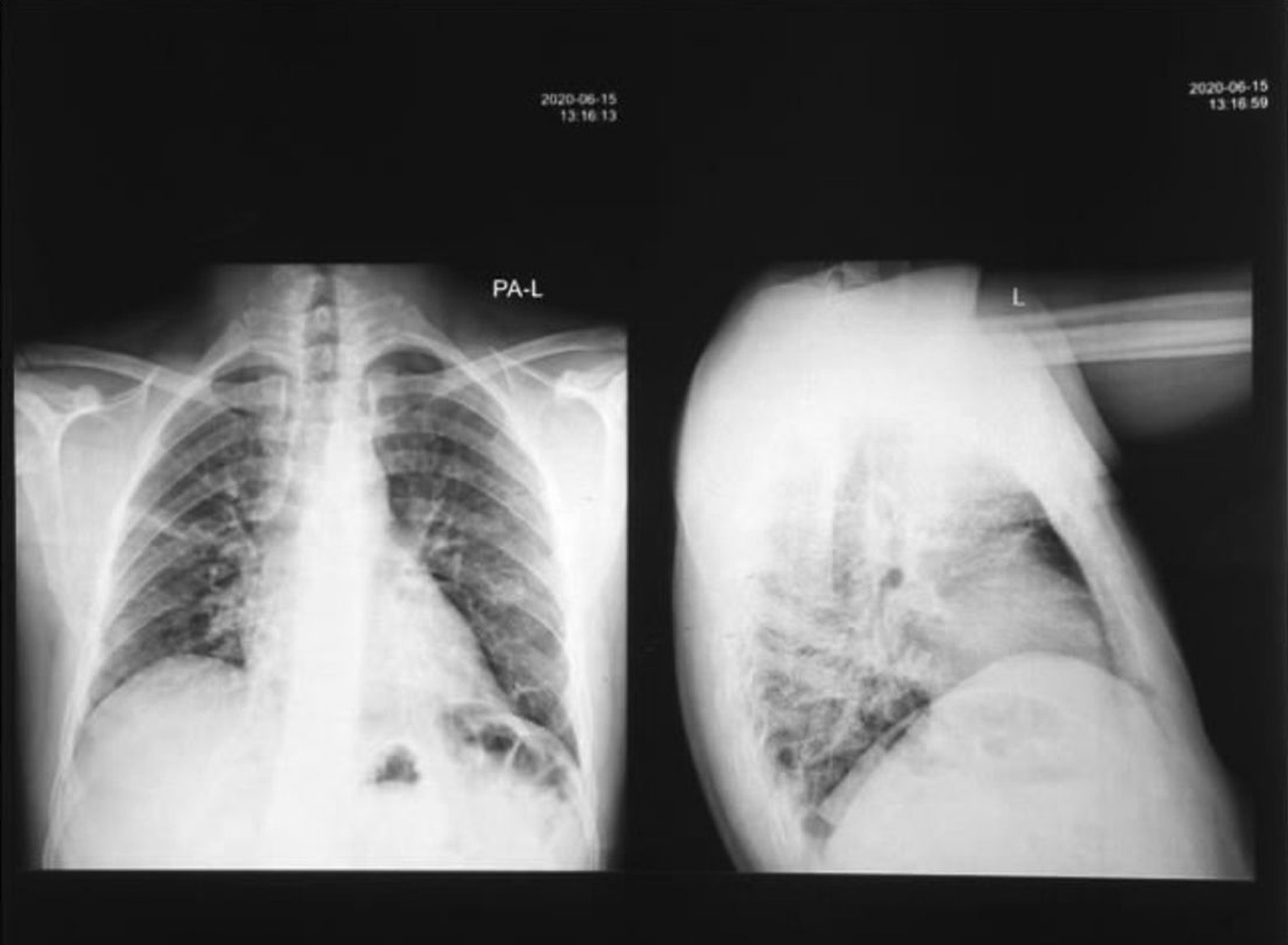
Anteroposterior/longitudinal chest X-ray (CXR AP/L). Imaging performed 10 days after COVID-19 presentation shows patchy infiltrates with ground-glass opacifications

A 32-year-old man from Yemen presented on 6 May 2020, with low-grade fever, sore throat, and insomnia. His father had passed away 5 days prior due to COVID-19. His medical history included a microadenoma that was diagnosed using magnetic resonance imaging (MRI) and secondary adrenal sufficiency, which was managed with steroid therapy. For 2 years preceding his diagnosis, the patient experienced daily headaches that were especially severe in the morning. They were usually located in the occipital area and were described as boring or occasionally throbbing pain. The headache increased with movement and stress, and sometimes responded to analgesics. In July 2019, the patient developed symptoms and signs of hypoglycemia. His random blood sugar level was 50 mg/dL during his first episode and 31 mg/dL during the subsequent episode. The patient also experienced general fatigue. His symptoms led to the diagnosis of secondary adrenal insufficiency, which was confirmed by low cortisol and cosyntropin levels. The patient was prescribed prednisone (20 mg/day). However, he did not receive any drugs or interventions for the pituitary microadenoma. Two months before the diagnosis of COVID-19, the patient developed blurry vision and scotoma in his left eye. He had normal thyroid function test results and normal prolactin levels. Antinuclear antibody test results were negative. The patient was a heavy smoker who smoked two packs per day. At the time of COVID-19 diagnosis, physical examination showed that the patient was febrile (temperature 38.3 °C) and acyanotic. His blood pressure, respiratory rate, pulse rate, glucose level, and oxygen saturation were 117/83 mmHg, 18 breaths per minute, 80 beats per minute, 118 mg/dL, and 95%, respectively. The patient was administered ceftriaxone (1 g twice daily intravenously), azithromycin (500 mg tablet once daily), and paracetamol (1 g tablet every 6 hours) but did not respond to treatment. Two days later, he became severely ill and toxic, and had symptoms of altered mental status, including hallucinations. He complained of shortness of breath (SOB) with progressive air hunger. Within the next 24 hours, his symptoms reached maximum severity. His oxygen saturation dropped to 80% on oxygen via a nonrebreather mask, and his temperature, respiratory rate, and pulse increased to 39.8 °C, 28 breaths per minute, and 95 beats per minute, respectively. Pulmonary physical examination revealed rhonchi and crepitation. Cardiovascular examination revealed no murmur, normal S1 and S2, and no gallops. No jugular vein distention was observed. Neurological examination revealed a Glasgow Coma Score of 15, fluctuating mental status, motor strength of 5/5 in all limbs, and intact sensation in all limbs. His abdomen was soft and nontender. No edema or rash was observed on the skin. The patient’s blurry vision and scotoma in his left eye increased in severity during the COVID-19 course. On the second day after the appearance of severe illness and dyspnea, the patient was administered methylprednisolone (80 mg twice daily intramuscularly for 5 days, followed by oral administration with dose tapering over 10 days until return to his daily dose of 20 mg). Three days after starting the medication, the patient’s health improved dramatically; the oxygen saturation increased to 91%, and fever, SOB at rest, and cough disappeared. On the ninth day after developing dyspnea, high-grade fever recurred, along with edema of the lower limb and abdominal discomfort. The patient did not have SOB at rest or cough, and his dyspnea gradually improved. Despite the administration of a variety of antibiotics, his fever did not improve. Chest radiography performed on day 10 (Fig. 1) showed some improvement in lung infiltration, but persistent ground-glass opacification. Repeat chest radiography performed on day 17 revealed significant improvement in lung infiltration and significant clearance of the ground-glass opacification. The patient remained febrile for 1.5 months. C-reactive protein (CRP) level during this period was normal. Erythrocyte sedimentation rate (ESR) was also normal during this period. Blood cultures revealed no growth, and malarial parasites were not observed on thin or thick films. With regard to his laboratory values, the patient’s white blood cell count increased throughout his disease course. Neutrophilia and lymphocytosis were also observed. The patient also had an increased number of monocytes. Later in the disease course, lymphopenia was observed. Platelet levels ranged from 293 to 90 g/L. The CRP level was elevated early in the disease course and later normalized. The ESR increased to 40 mm/hour and subsequently normalized. The D-dimer level peaked at 750 ng/mL, the ferritin level was 1250 ng/mL, and the lactate dehydrogenase levels ranged from 171 to 371 mg/dL. The troponin level did not exceed 0.008 ng/dL, and his creatinine levels remained normal. Immunoglobulin M (IgM) and IgG levels were 40 and 70 AU/mL, respectively. Chest radiography revealed bilateral patchy infiltration. Electrocardiography revealed normal sinus rhythm.

**Fig. 2:**
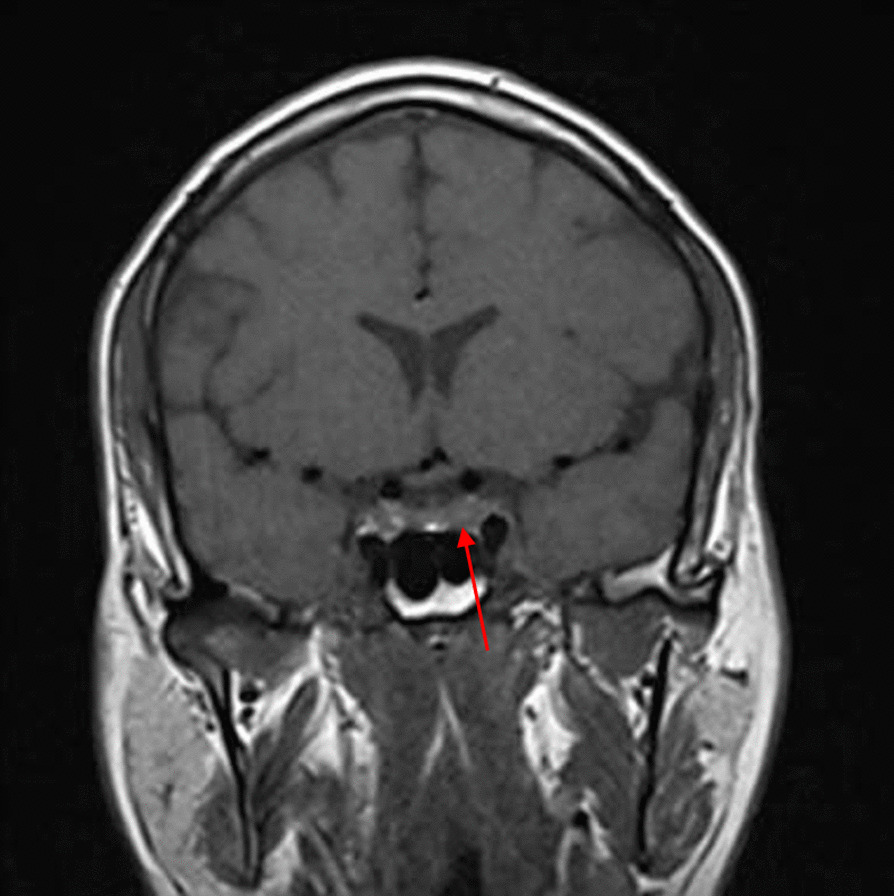
Pre-COVID-19 image of pituitary microadenoma. The image shows subtle asymmetry of both sides of the pituitary gland. The left side is slightly prominent, with a 3 × 3 × 2.5 mm faint ill-defined hypointense lesion (red arrow). Enhancement in the delay film shows no deviation of the pituitary stalk (microadenoma)

**Fig. 3:**
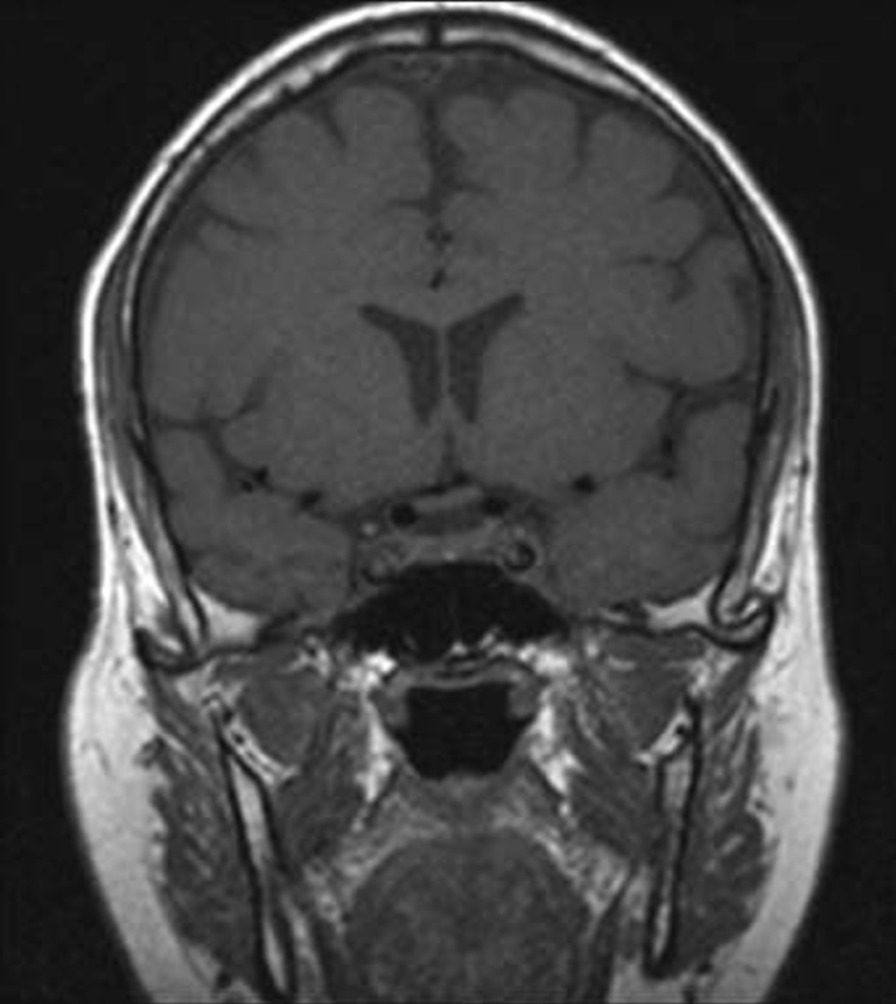
Post COVID-19 MRI findings. The image shows improvement of the pituitary microadenoma. The changes included the disappearance of the hypointense lesion and hyperintense enhancement on contrast observed on previous MRI. No macroadenoma or microadenoma is observed

**Fig. 4:**
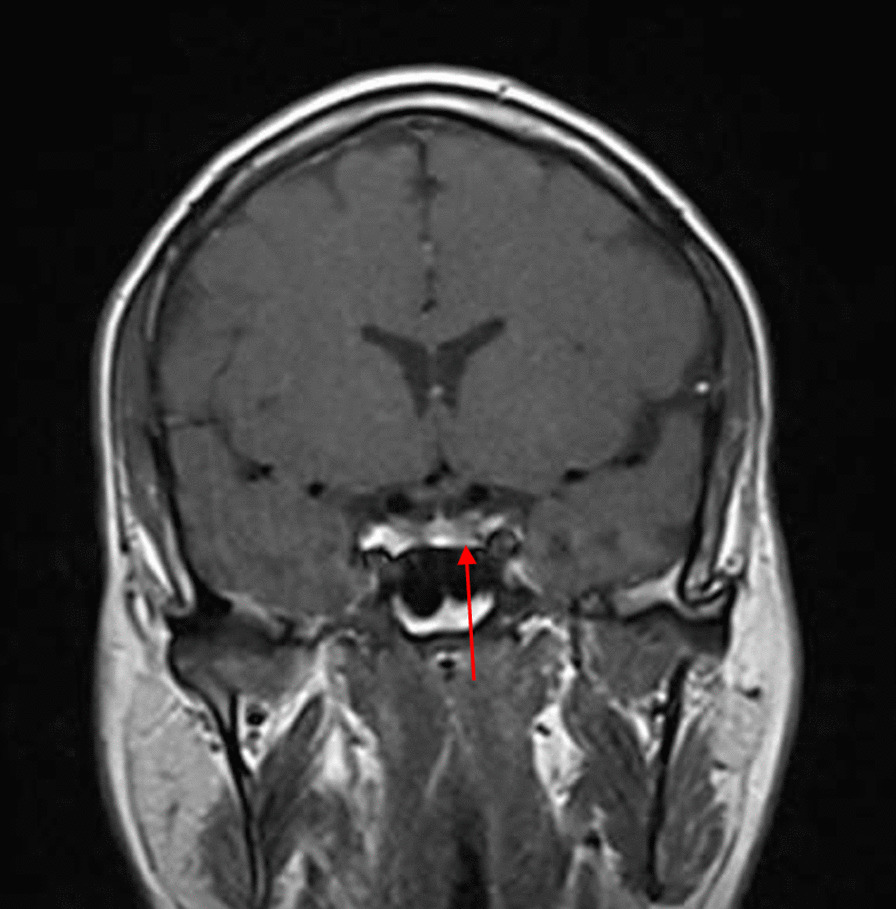
Pre-COVID-19 image of pituitary microadenoma. The image shows subtle asymmetry of both sides of the pituitary gland. The left side is slightly prominent, with a 3 × 3 × 2.5 mm faint ill-defined hypointense lesion (red arrow). Enhancement in the delay film shows no deviation of the pituitary stalk (microadenoma)

**Fig. 5:**
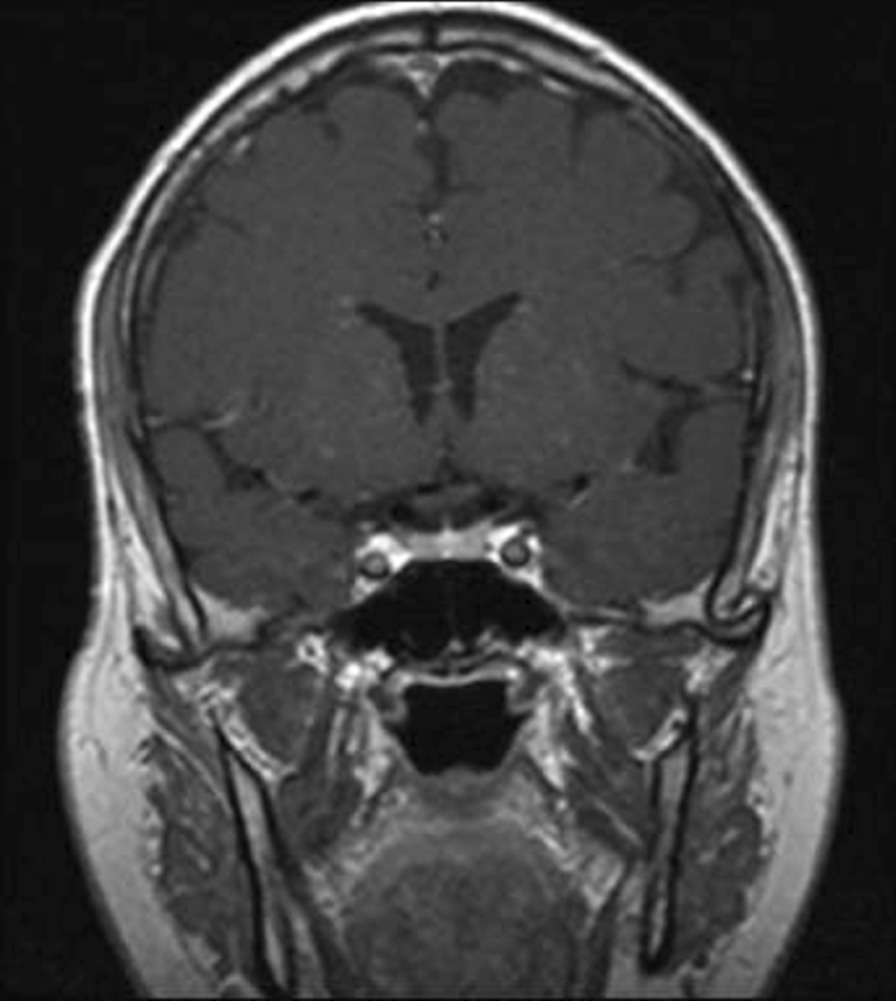
Post COVID-19 MRI findings. The image shows improvement of the pituitary microadenoma. The changes included the disappearance of the hypointense lesion and hyperintense enhancement on contrast observed on previous MRI. No macroadenoma or microadenoma is observed

On 18 August 2020, 3 months after the initial presentation to the hospital, the patient was advised to undergo a control brain MRI for comparison with his previous MRI findings (Figs. 2, 3). MRI showed improvement in the pituitary microadenoma. The changes included the disappearance of the hypointense lesion and hyperintense enhancement that was seen in the previous magnetic resonance images obtained on 28 March 2019. No macroadenoma or microadenoma was observed (Figs. 4, 5). Clinically, the patient’s blurry vision improved, and the severity of his headaches decreased drastically.

## Discussion

A growing field of research has suggested possible mechanisms for tumor resolution, including an acute infection leading to remission by inducing an immunological mechanism, which may have occurred in our patient, who developed COVID-19 [7]. A metalloproteinase, angiotensin-converting enzyme 2 (ACE2) acts as a receptor for severe acute respiratory syndrome coronavirus 2 (SARS-CoV-2), which causes COVID-19 [8]. ACE2 is highly expressed in the lungs, heart, gastrointestinal tract, kidneys, and bladder, with the lungs being the primary target of SARS-CoV-2. Once SARS-CoV-2 is bound, the spike protein undergoes protease cleavage [9]. As the virus attaches to ACE2 in the lung epithelium, macrophages, epithelial cells, and dendritic cells serve as the innate immune response until adaptive immunity is triggered. T-cell immunity has been studied in COVID-19 response [10]. At the site of infection, T cells produce cytokines, chemokines, and cytotoxic molecules. Cytokines directly inhibit viral replication, chemokines recruit additional cells to the site, and cytotoxic molecules directly kill the infected cells to eliminate the pathogen [11]. Memory T cells play a protective role after the infection resolves. Previous studies have demonstrated the role of virus-specific memory T cells in patients with respiratory diseases, including their correlation with protection during the recent epidemic caused by the H1N1 strain of the influenza A virus [11]. Many virus-specific CD4 and CD8 T cells remain in the body long after acute infection. For example, memory CD4 T cells specific for the HLA-DR08- and HLA-DR15-restricted epitopes within the S protein of SARS-CoV have been identified in recovered individuals, and respond to the similar SARS-CoV-2 [12]. Patients with COVID-19 show activation of the innate immune defense, leading to increased levels of circulating monocytes and activation of macrophages and antigen-presenting (dendritic) cells. These cells trigger the adaptive immune system (antibodies) along with natural killer cells (CRP elevation) to attack SARS-CoV-2. T lymphocytes are activated during the first phase of the disease and produce interferon gamma (IFN-γ). This response leads to an exaggerated immune response, damaging the lung tissue, and possibly selectively targets microadenomas. We hypothesized that two or more acute inflammatory responses occur simultaneously in patients with both tumors and inflammation caused by viral infection, ultimately negating the inhibitory effect of systemic inflammation on innate immunity. Thus, the activation of cells of the innate immune system, such as natural killer and dendritic cells, in patients with tumors results in spontaneous remission [8]. This case report presented the disease course of a man who presented with symptoms of COVID-19 and had a medical history of pituitary microadenoma that had been confirmed using MRI. The tumor went into remission after the patient contracted COVID-19, a change that was hypothesized to have occurred because of the induction of the innate and adaptive immune systems by viral infection. Remission progressed gradually, starting with improvement of the baseline symptoms of pituitary microadenoma, and remission of the pituitary microadenoma was confirmed using MRI (Fig. 3, 4). In the absence of specific treatment, remission may have been induced by COVID-19. Pituitary microadenomas rarely undergo spontaneous remission, as they are typically fixed in size and difficult to treat. PA, a reported cause of remission of a pituitary adenoma, was unlikely in the present case as it generally occurs in patients with macroadenomas and would have led to an empty sella along with symptoms of hypopituitarism, such as hypothyroidism or decreased libido, which were not observed in the present case. Other studies reported cases similar to ours and observed features similar to lymphocytic hypophysitis, an autoimmune disease in which the pituitary gland is infiltrated by lymphocytes, plasma cells, and macrophages, thereby impairing its function. However, the pathogenesis of lymphocytic hypophysitis remains unclear. Both autoimmune pathogenesis and viral origin have been suggested [1]; however, lymphocytic hypophysitis has characteristic features including symmetrical swelling, thickened pituitary stalk, and autoimmune disease. These features (except autoimmune disease and viral origin) were not present in our case with COVID-19-induced remission by immunity induction, which led to improvement in function [1]. A similar case report described remission of Hodgkin’s lymphoma after COVID-19. The authors hypothesized that SARS-CoV-2 infection triggered an antitumor immune response, which resulted in the cross-reactivity of pathogen-specific T cells with tumor antigens and natural killer cell activation by inflammatory cytokines produced in response to viral infection [2].

## Conclusions

We described a case of a pituitary microadenoma that regressed after the patient contracted COVID-19, suggesting a potential connection between inflammation and tumor resolution. The resolution of a pituitary adenoma is a rare occurrence, irrespective of whether it is related to the virus or a spontaneous event. We briefly reviewed COVID-19 pathogenesis and pituitary adenomas, and their clinical presentation. This case study may provide insights into the implementation of immuno-CoV-2 cancer vaccine therapeutic strategies for co-targeting tumors and COVID-19, as well as supporting cellular immunity to make hosts resistant to tumor development via inoculation with tumor site-guided agents. Further research is required in this emerging field to explore the connection between inflammation and cancer, which could have a ground-breaking impact.

## Data Availability

All data are included in the medical record of the patient. Clinical data are available from the corresponding author but only on reasonable request.

## Abbreviations

ACE2: Angiotensin-converting enzyme 2
COVID-19: Coronavirus disease 2019
CRP: C-reactive protein
ESR: Erythrocyte sedimentation rate
Ig: Immunoglobulin
MRI: Magnetic resonance imaging
PA: Pituitary apoplexy
SARS-CoV-2: Severe acute respiratory syndrome coronavirus 2
SOB: Shortness of breath
